# Youth athletes at Swedish sports high schools with an athletics specialism emphasise environmental support for injury risk management: a focus group study

**DOI:** 10.1136/bmjsem-2022-001527

**Published:** 2023-05-12

**Authors:** Jenny Jacobsson, Dejan Mirkovic, Per-Olof Hansson, Carolina Lundqvist, Robert Henry Mann, Ulrika Tranaeus

**Affiliations:** 1 Department of Health, Medicine and Caring Sciences, Linköping University, Linkoping, Sweden; 2 Athletics Research Center, Linköping University, Linköping, Sweden; 3 Swedish Athletics Federation, Stockholm, Sweden; 4 Department of Management and Engineering, Linköping University, Linkoping, Sweden; 5 Department of Behavioral Sciences and Learning, Linkoping University, Linköping, Sweden; 6 Children's Health and Exercise Research Centre, University of Exeter, Exeter, UK; 7 The Swedish School of Sport and Health Sciences, Stockholm, Sweden; 8 Sport Performance and Exercise Research & Innovation Center - Stockholm, SPERIC-S, GIH, Stockholm, Sweden; 9 Unit of Intervention and Implementation for Worker Health, Institute of Environmental Medicine, Karolinska Institute, Stockholm, Sweden

**Keywords:** Qualitative, Performance, Adolescent, Sporting organisation, Sporting injuries

## Abstract

In this study, we examined knowledge and understanding of sport-related injuries among youth athletics (track and field) athletes and assessed their needs in managing any health problems. Qualitative data were collected via 12 focus groups with youth athletes (16–19 years) studying at Swedish sports high schools with an athletics specialism. All focus group discussions were audiorecorded and transcribed before being analysed using a thematic analysis approach. Four researchers independently reviewed the transcripts, generated codes and developed themes. Three overarching themes related to the athletes’ knowledge and understanding of sport-related injury were developed: (1) awareness of injuries, (2) perception of injuries, and (3) factors contributing to injuries. The youth athletes were typically uncertain about how to acknowledge a sport-related injury. They expressed that knowledge about injuries was obtained in part by reflecting on the lived experiences of their peers. It was also demonstrated that there appears to be a ‘culture of acceptance’ regarding injury occurrence. In contrast, causes of injuries were viewed as dependent on multiple factors (eg, lack of context-specific knowledge about training practices). Regarding athletes’ needs in managing injuries, an additional three themes were developed: (1) creating functioning elite sports environments, (2) application of knowledge and (3) fostering athletes. An apparent lack of structure and organisation related to the school environment was identified as an important issue to review to create opportunities for sustainable athletic development. The study identified areas that can be improved in Swedish sports high schools with an athletic specialism and could be applied in other youth sports contexts. The results of this study guide school stakeholders, alongside the sport governing bodies who have the mandate to influence activities in youth sports contexts, whereby special attention should be directed towards improving the social environment for youth athletes.

**WHAT IS ALREADY KNOWN ON THIS TOPIC**

- Youth athletes studying at sports high schools are at high risk of sustaining injuries.

**WHAT THIS STUDY ADDS**

- Experiences of sport-related injury described by youth athletes highlighted uncertainty about how sport-related injury is acknowledged.
- There appears to be a ‘culture of acceptance’ regarding injury occurrence.
- Sport-related injuries were viewed as dependent on multiple factors (eg, lack of context-specific knowledge about training practices).
- A clear structure in athletes’ social environment was important for fostering sustainable athletic development.

**HOW THIS STUDY MIGHT AFFECT RESEARCH, PRACTICE OR POLICY**

- This study guides school stakeholders and sports’ governing bodies mandated to influence activities in these sports contexts.
- The proposed influence of environmental-level and individual-level factors on injury incidence suggests that a multilevel model (eg, socialecological framework) is useful for conceptualising the issue.

## Introduction

Studying at high school (ie, secondary education) represents a period when youth (adolescent) athletes are at a ‘development athletic level’ in most sports.^1^ This also represents a period in life when youth athletes are still under the responsibility and care of their parents/carers but when they also begin to make their own decisions about life and participation in sports.^2–4^ The opportunity to be a student at a sports high school (or sports academy) can facilitate the path towards a future career as an elite athlete.^1 5^ The overall aim of the sports high schools is to allow the students to have a ‘dual career’ by allowing them to combine secondary education and elite sports practice (eg, providing access to training facilities and time to practise their sport during typical school hours). Studying at this type of high school also means, in most cases, that youth athletes move away from home to a new environment. In Sweden, sport high schools are aimed at students aged between 16 and 19 who want to combine their secondary education with an elite sports education. These high schools—48 in total (representing 28 sports)—recruit nationally. That is, students from all over the country can apply. The education has a clear focus on elite sports. The Swedish school board approves the schools and is governed by the Swedish National Sports Confederation (https://www.rf.se). The affiliated national sports federation is the governing body for schools with a specific specialism.

Research from several countries has shown that sports-oriented school environments are not all positive and can contribute to unwanted consequences, such as health problems (ie, injury and illness) and burn-out.^6–9^ For example, the burden of injuries in such school settings has been reported to be high; 4 out of 10 athletes are injured during training periods, and close to 8 out of 10 athletes sustain a new injury during a calendar year.^6 8^ In addition, it has been shown that an athlete who begins a season with an injury is more than twice as likely, compared with non-injured athletes, to sustain additional injuries during the season, which will greatly affect the athlete’s ability to participate.^8^

Research in Swedish Athletics (SA) and at Swedish Sports High Schools, with an athletics (track and field) specialism (SSHS-A), has presented evidence that 16-year-old athletics athletes have almost the same prevalence and incidence of injury as older adult elite athletes over a full athletics season and that most injuries are related to overuse.^6 10^ Although these observations have been well documented, the literature is sparse on the underlying causes of the high injury rates in these sports-orientated school environments. This could be because most research has adopted prospective and longitudinal designs, almost exclusively focused on quantitative outcomes. Although useful, this approach will not provide a full and in-depth understanding of the context of existing problems.^11^ Also, research with youth athletes that has used mixed methods or qualitative approaches has shown that complex causal relationships (eg, between sleep and training volume) may exist.^12^

As the governing body, SA is ultimately responsible for how the activities are conducted at the SSHS-A. To develop tailored interventions addressing ill health in these settings, SA needs to gain a deeper overall understanding of the reported problems from the stakeholders’ perspective of the reported problems.^13^ The need to supplement quantitative study designs with qualitative approaches has been emphasised.^14^ Moreover, this research project is also supported by previous research which emphasises the importance of involving end users (ie, youth athletes) early in the development and implementation of health programmes (eg, introducing measures to reduce injury risk) and that these programmes are contextualised to the specific age and social environment of the athletes.^15 16^ Because musculoskeletal injuries have previously been identified as a significant problem in this group of athletes,^6^ the initial objective of this study was to examine the knowledge and understanding of musculoskeletal injuries and their occurrence among students at SSHS-A. A second objective was to understand athletes’ needs in managing health problems in this setting.

## Methods

### Design

This was a qualitative study using a descriptive exploratory approach. Semistructured focus groups were used for the data collection because we aimed to generate discussion among the athletes, thereby facilitating context-specific and in-depth insight. In this nationwide study, data were collected in six SSHS-A, including first- and third-year students (year 3 is the most students’ last year in high school). The analysis results are reported according to the Consolidated Criteria for Reporting Qualitative Research (online supplemental material).^17^

10.1136/bmjsem-2022-001527.supp1 Supplementary data

### Participants

Schools were recruited by invitation from the Education Officer at SA. The director for the athletics programme at each SSHS-A (n=7) was first contacted by telephone. Then additional emails were sent to each high school approximately 4 weeks before each focus group. These emails described the purpose of the project and the procedures for the focus groups. One of the SSHS-A declined participation because the director in charge was ill. The director at each of the SSHS-A then informed the students about the purpose of the study, and they were subsequently invited to participate. We aimed to include all first-year and third-year student athletes studying at the SSHS-A, and, therefore, a purposive sampling method was used.^18^ Participation in a focus group was voluntary, and athletes could refrain from participating without providing a reason. No parental consent was required due to the age of the students. All participating students consented to participate in the study and for the focus groups to be audio recorded.

### Data collection

Data were collected from 12 focus groups, including 6 in each cohort of students (ie, first-year and third-year students). Attendance ranged from 5 to 15 participants, and all focus groups took place between October and December 2018 in an auditorium at each SSHS-A. The mix of girls and boys was fairly even in each focus group, whereby the total number of participants per group mainly depends on whether the school is located in a larger or smaller town. Participant information is presented in table 1. Two representatives from the research group were present at all focus groups; one moderated the discussions (JJ; medical coordinator for SA, physical therapist), while the other recorded the focus groups and acted as a facilitator for the discussions (DM, head of dual careers at SA). The researchers’ collective knowledge and expertise were useful in building relationships and facilitating athletes who wanted to share their lived experiences. All focus groups followed the same standardised protocol based on a semistructured topic guide (see online supplemental material) and lasted for approximately 60 min. Each focus group began with a short introduction of all participants and was followed by a 5 min report of scientific findings from previous injury research conducted by SA and at SSHS-A.^6 10^ Each focus group was divided into two parts. First, the participants were asked to reflect on and discuss what could be the cause of the high incidence of injuries (ie, a health problem) at SSHS-A. In the second part, the athletes were asked to reflect on what needs to be done to reduce health problems and how a change can be achieved. Every focus group (including facilitator-participant interactions) was unique; different prompt questions were used to encourage in-depth responses. All focus groups were audiorecorded and transcribed verbatim after the final focus group. Focus group transcripts were then circulated to four researchers in the team (JJ, DM, P-OH and UT), the latter two with experience in psychology, pedagogy and qualitative research. All audio files were permanently deleted after transcription.

**Table 1:** Description of the participants

Focus group participants	Year 1 students (n=74)	Year 3 students (n=45)
Female, n (%)	39 (53)	22 (49)
Age (years), median (range)	16 (16–17)	18 (18–19)

### Data analysis

Data collected from the 12 semistructured focus groups were used in the analysis. The analysis started after the last focus group, and investigator triangulation was used to support the conclusions.^19^ The data from the focus groups were analysed inductively (ie, directed by the content of the data) using a thematic analysis approach.^20^ This six-stage process enables data to be developed into codes and themes. In the first stage, the transcripts from all focus groups were reviewed independently by four researchers (JJ, DM, P-OH and UT) who familiarised themselves with the data. Two of the authors (JJ and DM) at the focus groups used a latent approach (ie, identifying underlying ideas/assumptions) when analysing the transcripts. The second and third stages included individual attempts to code and develop these codes into themes. In the fourth stage, all four researchers compared and discussed the codes and themes at a face-to-face meeting. Any discrepancies between the independent coding and themes were discussed in the fifth stage until a group consensus was reached. The sixth and final stage involved writing up the data. The agreed codes and themes were then reviewed independently by two researchers (CL and RHM) who had not taken part in the study design or initial data analysis. This process was used to ensure that the codes and themes were appropriately challenged and understood according to this study’s objective(s).

### Results and discussion

Regarding the first objective of the study, to examine youth athlete’s knowledge and understanding of musculoskeletal injuries in the sport, the following three themes were generated from the focus group discussions: (1) awareness of injuries, (2) perception of injuries and (3) factors contributing to injuries (table 2). Further, three themes were generated regarding the second objective of the study, which describes athletes’ needs in managing injuries (table 3), as follows: (1) create functioning elite sports environments, (2) application of knowledge and (3) foster athletes. Overall, these themes show that transitioning from being a talented youth athlete to having a career as an elite adult athlete is a complex path with several possible obstacles, as supported by previous literature.^21 22^

**Table 2:** Athletes’ perceived knowledge of injuries

Themes	Codes	Subcodes	Example of quotes from youth athletes
Awareness of injuries	Experiences of injuries	Ambiguity	‘Yes, but I think, of course, I’ve had niggles, it’s not that, but I’ve never gotten so far that I’ve really thought there were injuries.’ Year 3
			‘I would probably draw the line at when you can’t exercise, then it’s a big injury. When you lose training.’ Year 3
	Others’ injuries	The experience of others	‘There are many who have been injured, so you have an idea.’ Year 1 ‘Everyone has had some experience with an injury.’ Year 3
Perception of injuries	Intrapersonal perspective	Concern	‘Well, I went around and worried for a long time. That’s probably why I hesitated when I applied here at all, because like what am I going to do here if I’m in ‘rags’. And then when you didn’t get a hundred either, you were a little disappointed too. I had it as a goal, like, you have to be 100 when you start here.’ Year 1
			‘You get a little worried if you feel pain you haven’t felt before, in a place you haven’t had pain before. So, you think, what is it? Should I continue, should I not continue?’ Year 3
	Interpersonal perspective	Culture, acceptance	‘Track and field athletes have niggles all the time, somewhere. Especially sprinters.’ Year 3
			‘Heard that those who are the best at fighting through injuries are the ones who become the best. Everyone gets hurt at some point.’ Year 1
		Lack of medical (social) support	‘So, my experience is that the physiotherapist who is connected to the SSHS-A has a lot to do, so you don’t really get the time that you really need.’ Year 3 ‘It’s very much an ‘assembly line’, in with the next one, out with that one and so on. It’s like that.’ Year 3
		There is social support	‘…talked to him who is the coach here. He has like all the time asked how my injury is going and he’s been involved in it, every time we have met each other. So, I have, of course I’ve been a bit worried, but he’s always known about it and sort of cared.’ Year 1 ‘If you feel that that was not good, then you just tell them.’ Year 3
Factors contributing to an athletics injury	Lack of context-specific knowledge	Inaccuracy	‘I feel a lot in the gym, if you do exercises wrong. It is important to know from the beginning that you are doing them correctly, because if you do it wrong and then you continue, you become uneven and so on.’ Year 3 ‘No, that was the whole first year, meatballs and sausages that was all you ate.’ Year 3
		Training structure	‘Before, I trained maybe 3, 4, max 5 times a week. Say running then, maybe not that I ran every time but that I trained athletics. And then when I started here, it increased quite a bit. Training 2 times in the same day and stuff like this, things I hadn’t done before. It wasn’t impossible, that you got up 7, 8 sessions a week and so on. Of course, you had a day off. But in any case, it was a big increase compared to what we did before, and the sessions became longer, and to some extent more intense as well. And that was something I, I wanted to invest in too, and then it turned out that you have to increase the amount of training. Maybe just too much for my body.’ Year 3
			‘Or too much training, if you run several sessions a day. Kind of like 2.5 hours.’ Year 1
			‘After all, we always train on fairly hard surfaces, athletics tracks.’ Year 3
	Biopsychosocial	Lack of recovery	‘That there are relatively few elements, if you do one event in athletics, you might be training a little one-sided, I think.’ Year 3 ‘It’s more fun to stay up and talk with your friends than to go to bed.’ Year 1
		Structure every day	‘Then, sometimes it feels like you eat too little and I’ve always eaten too little, like.’ Year 1 ‘…you have to plan a lot. Come home at 8 pm and start cooking, even though you don't really have the energy.’ Year 1
		Lack of social support	‘It is difficult for coaches to know how injured we are.’ Year 3
			‘If I think I need to do my rehab, then there shouldn’t be anyone who sort of argues against it. I think that before you set up the week, that is, those who plan, that is, the coaches at and those at home must have some form of communication so that they are reasonably in sync. But if I have to train differently, I think that I should be able to do that, and it shouldn’t be strange that I do.’ Year 3

**Table 3:** Athletes’ description of their needs for injury management

Themes	Codes	Example of quotes from youth athletes
Create a functioning elite sport environment	Social support	‘I think that what is a little worse for us who attend elite sports, is that we never have a support session, for example, at school. We will never, I mean we never have time to go to those sessions because, because we have often training during them. We have training every afternoon, almost every afternoon and that is usually when they add these support sessions, and we have such a busy schedule. So, I’ve never been able to go for any support. It has been difficult getting the extra help you need at school, I’ve thought about that. I had X and he really addressed this with my performance anxiety so I got help with it, the SSHS-A paid for me to go and see a sports psychologist, I’m very grateful that I got to do it and that X really took it seriously. Because it was very extreme when I was at my worst. So, I think that’s great, but it’s also like this, X is, he’s like a father type.’ Year 3
	Medical support	‘There is quite a lot of pressure on them. It feels like, yes this week we are busy and next week too but come back then.’ Year 1 ‘If you have niggles at all the training sessions and at competitions, then you have to talk it through with someone.’ Year 3
	Coach support	‘Together with her, I came to the conclusion that you study until 9 and then you stop. And it makes me sleep a lot better, because I can relax like… so she’s more of a mentor than a coach.’ Year 3
		‘….for a while I chose only to do rehab, I had a really bad lower back. Then the coaches talked to each other and said ‘but ok, she will do her rehab every morning session and then she won’t be part of the joint training.’ Year 3
	School support	‘Here you get to think about the whole, breakfast, schedule, etc. The entirety of training for athletics.’ Year 3 ‘It was a big change. It became difficult with great stress at school and stress with training.’ Year 1
Application of context-specific knowledge	Preventative measures	‘As a runner, I check the number of miles a week, for example, to make sure I don’t run too much.’ Year 3
		‘But like if you are not prepared enough to do anything. And then you start doing something a lot. The body has to get used to the new adjustment. The body somehow can’t cope with changing, changing and doing new things so quickly.’ Year 1
		‘Screening—because if you are not injured, it is difficult to think that it is preventive.’ Year 1
	Own responsibility	‘… I sort of have a programme that I will do throughout my career. And I don’t have problems with my hamstring anymore, it’s kind of good, and I have, I think it’s because I kind of do it, basically every day.’ Year 3
		‘But that’s fine, at least I’ve learned when I’ve been injured, I’ve been injured for a few years, so I’ve gotten to know my body better. That it’s okay to skip training because it hurts, because it’s mental that it’s hard to skip training, watch others run. You might not think what the consequences will be if you train, it might be that you have to stand aside for a longer time.’ Year 3
Fostering athletes’ education	Need factual knowledge	‘I would like a little more information about what we should eat. I had no idea what was good for me and what I should eat to get energy.’ Year 3 ‘As a runner, I check the number of miles a week, for example, to make sure I don't run too much.’ Year 3
	Structure every day	‘I feel like I was a rambunctious little boy when I started. I didn’t know about prevention or anything like that and I feel like I’ve become stronger and aware of what I need to do to not get injured and to get better.’ Year 3 ‘…you have to get into routines, you have to do the dishes, cook, wash, clean. So, I think that’s why many people maybe cook a little worse food in the first place. You might not have the time, because you have to train, you have to study and you have to do everything.’ Year 3
	Diet, sleep	‘If they had tips on dishes, so that you don’t just go through the whole first year frying meatballs and sausages.’ Year 3 ‘Sleeping is difficult.’ Year 1

### Athletes’ perceived knowledge of sport-related injuries

#### Awareness of injuries

The definition of an injury can be viewed from various perspectives; for example, sports impairments rely on accurate athlete self-reporting of health problems.^23^ The youth athletes at SSHS-A displayed uncertainty about how to acknowledge an injury in the sport and when they should consider that they are injured. As one athlete expressed, ‘*I’ve had niggles, it’s not that, but I’ve never gotten so far that I’ve really thought there were injuries.’* Similar findings have been observed in other sports environments among athletes of the same age.^6 24^ In addition, these youth athletes expressed that, in part, knowledge about injuries was obtained by reflecting on injuries that their peers experienced; ‘*There are many who have been injured, so you have an idea.’* This uncertainty shows that the athletes may lack certain important information to be able to perform their sport (eg, on how to interpret body signals). A preventative behaviour has been described as developing over time, related to own injury experience.^25^ However, from the view of the sport and key stakeholders, it should be considered unacceptable that young talented athletes must experience periods with injuries to provide them with the tools necessary to deal with any future injury problems.

#### Perception of injuries

Doubts were expressed about applying to SSHS-A if one did not feel physically prepared. As one athlete put it: ‘*The body was not 100 percent.’* This signals that when athletes begin their studies, they may believe that the focus of SSHS-A is primarily on physical performance and that they do not fully comprehend the purpose of dual career education. Such reasoning is problematic, and sports federations and schools need to identify and understand these athletes to provide the necessary support to avoid the risk of talented athletes dropping out.^26^ Furthermore, expressions from the focus groups indicate that there appears to be a well-embedded culture of acceptance that injuries will occur within the sport. If such a culture exists, one consequence is that experiential pain is ‘normalised,’ that is, the young athlete may not understand or even dare to express that they have a problem.^27^ As a result, youth athletes may be training and competing while experiencing a health problem.^28^ The problems that can arise due to a hidden cultural adaptation (ie, ‘silent issue’) have been highlighted as important to identify so the situation can be remedied.^29^ In addition, athletes at some SSHS-A experienced difficulties in receiving proper support for perceived health problems, exemplified by the following quote: ‘*It’s very much an ‘assembly line,’ in with the next one, out with that one, and so on’*. If they received support, this was described as being strongly dependent on, for example, the coaches’ personal experiences rather than an overall structure in the school environment as put by one athlete: ‘*He has like all the time asked how my injury is going’*.

#### Factors contributing to injuries

Training structure and its possible relationship with injuries was widely discussed in all focus groups. The athletes expressed a lack of context-specific knowledge and experience of a one-sided structure, for example, about meal planning and how training is conducted: ‘*It is important to know from the beginning that you are doing them (exercises) correctly […].’* The reflections of the athletes show a need to acquire in-depth knowledge early in their sporting and academic careers about the specific loads and possible risks in their sport.

The biopsychosocial model describes that an individual’s health or ill health can depend on biological, psychological and social aspects that interact to varying degrees.^27^ One athlete’s statement, ‘*If I think I need to do my rehab, then there shouldn’t be anyone who sort of argues against it […]’,* reflects that athletes may experience stress or frustration in having to explain the situation and experience inadequate communication with/between coaches, instead of being supported by them. The latter highlights the importance of a closer dialogue with (and support from) coaches to enable the athlete to navigate health risks better. Overall, in line with what has been observed previously, this research suggests that the causes of sports injuries are dependent on multiple factors.^12 27^

### Athletes’ description of their needs for injury management

#### Create functioning elite sport environment

The environment in which the athletes find themselves and how it can affect performance, well-being and longevity in the sport has been emphasised in several studies.^5 30–32^ In our study, the athletes’ perceived lack of structure and organisation related to the school environments was discussed in the focus groups. It emerged as possibly the most important issue to review to create opportunities for sustainable sports development. For example, the athlete’s description of a lack of opportunity to receive support for academic studies during scheduled school time*—‘we never have time to go to those sessions because, because we have often training during them’*—reveals that some schools may not fully grasp the extent of the athlete’s studies and training routines. This points to an important area that should be straightforward for schools to address, for example, when planning students’ schedules. The purpose of SSHS-A environments is to support the athlete’s personal development and provide opportunities for a dual career. The SSHS-A’ holistic approach was emphasised by the athletes as important for their study time at the school. In addition, perceived shortcomings in the school networks were also discussed, such as difficulties in accessing various resources (eg, physiotherapists, dietitians and sports psychologists) when needed. Thus, there is a need for SSHS-A to review the various collaborations so that school-related resources that are to be provided are available in a similar way regardless of which school one attends, regarding, for example, accessibility.

#### Application of context-specific knowledge

There is an assumption that a certain level of knowledge (eg, risk literacy) should exist for an understanding of a problem to exist.^15^ The athletes often described how individualised programmes and advice provided increased understanding that helped them manage their training and injuries: ‘*As a runner, I check the number of miles a week, for example, to make sure I don’t run too much.’* The importance of factual and conceptual knowledge to understand complex relationships and take personal responsibility for preventing health problems in athletes has been highlighted.^22 33 34^ This highlights that the sports subjects on the schedule may need to be revised so that, in addition to the basic generic information, they also contain sport-specific knowledge that the athletes can relate to.^35^

In addition, the athletes’ lack of understanding of why, for example, special exercises (resulting from screening) are introduced as prevention shows that existing models for knowledge transfer do not seem to work. Knowledge translation refers to how complex information is translated and made understandable and useful to intended consumers. Actions that support both knowledge translation and delivery have been identified.^36^

#### Fostering athletes/education

The concept of learning is a process that can be problematised in a broader sense and includes the need for informal learning alongside purely theoretical formal learning.^37^ Contextual, informal or formal learning can transform knowledge into a structure of understanding to support athletes’ self-management.^13 37^ To avoid and manage the consequences of health problems, the results of this study indicate that SSHS-A should have a clear structure for individually adapted study and training plans that are regularly monitored and evaluated for each athlete. Such an understanding (ie, taking an individualistic approach) supports the development of life skills and should enable youth athletes to balance their dual career opportunities.^26 38^

In summary, several needs have been identified that need to be met to give students at sports high schools better conditions to deal with possible injuries. Future measures should be aimed at multiple levels, ranging from how the Sports High School is organised with, for example, supporting network (organisational level) to provide the individual athlete with factual knowledge that increases his understanding of the complex relationships around injury management (intrapersonal level). This emphasises that a socioecological framework is useful for planning future interventions.^39^

### Methodological considerations

While a qualitative study design is a useful approach for exploring how the problem of injury is experienced and defined by youth athletes, various methodological considerations need to be discussed. For example, when framing generalisability from a ‘statistical-probabilistic’ perspective, the results of this study should be viewed as being context-specific, and caution should therefore be applied if looking to generalise the findings to other age groups and/or contexts outside of the Swedish and/or Scandinavian high school model. The investigator triangulation method used in this study supports the trustworthiness of the findings. This claim of trustworthiness, in this case transferability, is also supported by the fact that a degree of similarity was demonstrated between the findings presented in this study and previous literature. We believe that the researchers’ expertise combined with their different backgrounds enabled a rigorous analysis process, and resulted in findings that comprehensively describe the data collected. Transferability was met by including quotes in tables 2 and 3. However, as with all qualitative research, the researcher’s own experiences and views influence the interpretation of the data—upheld here as a positive element of the study.

## Conclusion

The process of compiling the youth athletes’ experiential knowledge showed that the journey from talent to the elite is influenced by several factors, some of which are seemingly particularly important to consider to achieve good health during academic studies at SSHS-A. The proposed influence of environmental-level and individual-level factors on injury incidence reported in this study suggests that a multilevel model, for example, a socialecological framework, is useful for addressing the question at issue, protecting the athlete’s health.^21 40 41^ Several proposed areas that could be implemented in the SSHS setting emerged during the focus groups. The results of this study thus guide school stakeholders and sports governing bodies who have the mandate to influence the activities in these sports contexts, whereby special attention should be directed towards actions in the athlete’s school social environment. The needs described are based on the athlete students’ experiences. Therefore, future action programmes and strategies implemented within SSHS-A will likely have the support and be taken up more effectively by intended users.

## Data Availability

No data are available.
