# Supplementary material for: Youth athletes at Swedish sports high schools with an athletics specialism emphasise environmental support for injury risk management: a focus group study
Source: BMJ Open Sport Exerc Med. 2023 May 12;9(2):e001527. doi: 10.1136/bmjsem-2022-001527 (PMC10186414; doi:10.1136/bmjsem-2022-001527)
Supplement: Supplementary data [file bmjsem-2022-001527supp001.pdf]

Youth athletes at Athletics (Track and Field) Swedish Sport High Schools emphasize the need for environmental support for injury risk management: a focus group study. *Jenny Jacobsson, jenny.jacobsson@friidrott.se*

Interview guide for focus group meetings at athletics national high sports schools

INTERVIEW GUIDE, 60 minutes

Warm up

Presentation of participants.
Reflections regarding the presentation of studies performed in Swedish Athletics and at the athletics national sport high schools. Is it a familiar situation described? Do the participants recognize a potential problem with injuries?

Part 1

The students' experiences of what the relatively high incidence of injuries may be caused by and what can be done to reduce injuries among students at national high sport schools.
Purpose:
Catch the students' perceptions and experiences about the causes and measures to reduce injuries.
Trigger questions:
• Why do you think that there are so many injuries among students at national high sport schools?
• What do you think may be the cause? Reasons?

Part 2

The students' thoughts on how a change will come about.
Purpose:
Catch the students' experiences about what can be done to bring about a change. How this will be done in practice.
Trigger questions:
• What do you think can be done to reduce injuries?
• How do you think this can best be achieved?
• What changes do you think we should make first?
• Can you give some concrete proposals on what should be done?
